# Radiological imaging features of the salivary glands in xerostomia induced by an immune checkpoint inhibitor

**DOI:** 10.1007/s11282-020-00480-9

**Published:** 2020-09-07

**Authors:** Kouji Katsura, Saori Funayama, Kayoko Ito, Kaname Nohno, Noboru Kaneko, Masaki Takamura, Marie Soga, Taichi Kobayashi, Takafumi Hayashi

**Affiliations:** 1 Department of Oral Radiology, Niigata University Medical and Dental Hospital, 1-754 Asahimachi-dori, Chuo-ku, Niigata City, Niigata 951-8520 Japan; 2 Oral Rehabilitation, Niigata University Medical and Dental Hospital, 1-754 Asahimachi-dori, Chuo-ku, Niigata City, Niigata 951-8520 Japan; 3 Department of Preventive Dentistry, Niigata University Medical and Dental Hospital, 21-754 Asahimachi-dori, Chuo-ku, Niigata City, Niigata 951-8520 Japan; 4 Division of Preventive Dentistry, Faculty of Dentistry and Graduate School of Medical and Dental Sciences, Niigata University, 2-5274 Gakkocho-dori, Chuo-ku, Niigata City, Niigata 951-8514 Japan; 5 Division of Oral and Maxillofacial Radiology, Faculty of Dentistry and Graduate School of Medical and Dental Sciences, Niigata University, 2-5274 Gakkocho-dori, Chuo-ku, Niigata City, Niigata 951-8514 Japan

**Keywords:** Diagnostic imaging, Salivary gland, Xerostomia, Adverse drug event, Sjögren’s syndrome

## Abstract

The clinical features of xerostomia induced by immune checkpoint inhibitors (ICI) are similar to those of Sjögren’s syndrome (SS), whereas the immunohistological and serological features are known to differ from those of SS. However, the radiologic imaging features of salivary glands are not yet well known. We report a case of a 56-year-old male patient diagnosed with ICI-induced xerostomia. The patient underwent various imaging examinations to investigate the condition of the salivary glands, which indicated the following: (1) less specific findings on contrast-enhanced computed tomography, (2) mixed with intermediate and low signal intensity on both *T*_*1*_-weighted and fat-suppressed *T*_*2*_-weighted magnetic resonance imaging and poor “salt and pepper” appearance on magnetic resonance sialography, and (3) multiple ovoid hypoechoic areas with hyperechoic bands without acute sialadenitis on ultrasound. These radiologic imaging findings suggested remarkable lymphocyte infiltration, which could be a characteristic of ICI-induced xerostomia.

## Introduction

To date, immune checkpoint inhibitors (ICI), including ipilimumab or nivolumab, are used for the treatment of various types of cancers. The mechanism of ICI against cancer cells is augmentation of the anticancer immune response by blocking the negative costimulation of T cells [1]. By contrast, ICI can induce many kinds of hyperimmune reactions, categorized as immune-related adverse events in normal organs. To date, lichenoid reactions, xerostomia, and dysgeusia have been the primary reported oral adverse events [2–8].

The mechanism of ICI-induced xerostomia is considered as an impairment of the PD-1/PD-L1 pathway caused by ICIs that trigger the activation of T-lymphocytes, leading to infiltration of the salivary gland epithelium [8, 9]. As a result, it is suggested that there is a lack of the salivary acinar cells and salivary dysfunction is caused [10].

The clinical characteristics of ICI-induced xerostomia are similar to those of Sjögren’s syndrome (SS)-induced xerostomia, whereas the immunohistochemical and serological features are known to differ from those of SS [6–8]. However, the radiologic imaging features of the salivary glands are not yet well known. Particularly, to our knowledge, there have been no published reports of salivary gland imaging using computed tomography (CT) or magnetic resonance imaging (MRI), although some studies have reported the use of ultrasonography (US) for this purpose [6, 8]. We present a case of nivolumab-induced xerostomia in which we obtained radiologic imaging findings of the salivary glands using US, CT, and MRI. This case report provides the imaging findings for a novel immune-mediated sialadenitis caused by ICI that is radiologically distinct from SS-induced xerostomia.

## Case report

A 56-year-old male patient with bilateral renal cancer, diagnosed as clear cell carcinoma, received nivolumab (240 mg/day) for renal cancer treatment at the affiliated hospital of Niigata University Medical and Dental Hospital. The patient had type 1 diabetes and diabetic renal failure. He had no subjective or objective signs of xerostomia until the fifth course of nivolumab, after which he complained of dry mouth and dry eye. After the fifth course of nivolumab, his serum total amylase level increased to 645 U/L (normal range: 40–130 U/L), with the serum salivary amylase level elevated at 611 IU/L. Subsequently, his amylase level decreased markedly to below the normal range (7–35 U/L). Based on the patient’s clinical course and laboratory results, he was diagnosed with nivolumab-induced xerostomia.

**Fig. 1:**
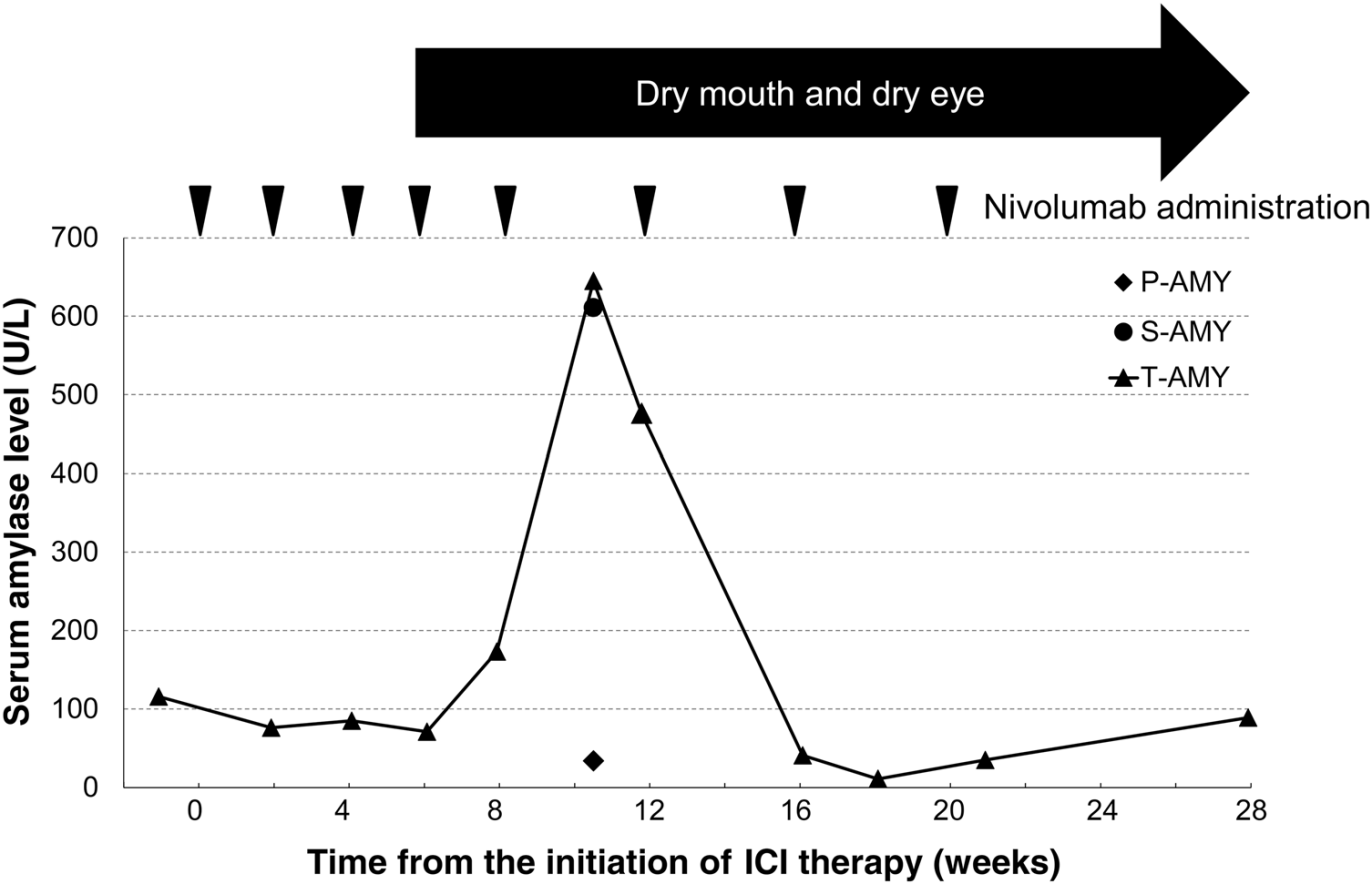
Relationship between the changes in serum amylase level and the clinical course. The *X*-axis indicates the weeks elapsed after the initiation of nivolumab. The *Y*-axis indicates the serum amylase level (U/L). The arrowhead indicates the day of nivolumab administration

**Fig. 2:**
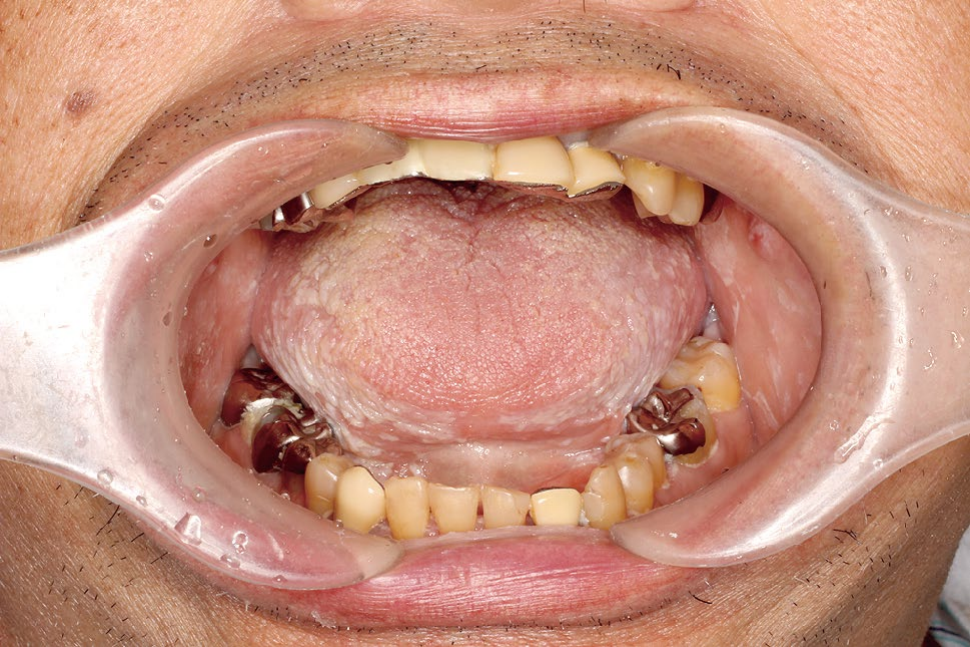
Saliva was not observed in the mouth, and oral candidiasis-like white patches were recognized on the ventral surface of the tongue and the surfaces of the bilateral buccal mucosa

After the eighth course of nivolumab, ICI therapy was discontinued because of consciousness disturbance and acute hepatic dysfunction. The patient was transferred to the Department of Urology, Niigata University Medical and Dental Hospital, and to treat these immune-related adverse events, corticosteroids were initiated, consisting of intravenous prednisolone 60 mg/day (1 mg/kg/day). Disturbance of consciousness and acute hepatic dysfunction improved rapidly after the start of corticosteroids. Two months after discontinuation of ICT therapy, the total serum amylase levels normalized (89 U/L). Figure 1 shows the relationship between the changes in serum amylase levels and ICI therapy. However, there was no improvement in dry mouth or dry eye symptoms. Serological test, the Saxon test, and the Schirmer test were conducted 2 weeks before, 2 weeks after, and 2 months after discontinuation of ICI therapy, respectively. The serological test results were negative for both anti-SS-related antigen A (anti-SSA) and anti-SS-related antigen B antibodies. The amount of stimulated saliva by Saxon test was 0.32 g/2 min, and the Schirmer test produced tears of 3 mm/5 min for the right eye and 2 mm/5 min for the left eye. These results showed that salivary and lacrimal functions were decreasing markedly, although the results of the serologic test were negative for SS (Fig. 2). Lip biopsy was not performed because SS was excluded by the clinical course and the serological test results. This patient was referred to the Department of Oral Radiology, Niigata University Medical and Dental Hospital, for detailed imaging examination and imaging diagnosis of the salivary glands. US, contrast-enhanced CT, and MRI were conducted at 2, 3, and 9 weeks after discontinuation of ICI therapy, respectively.

**Fig. 3:**
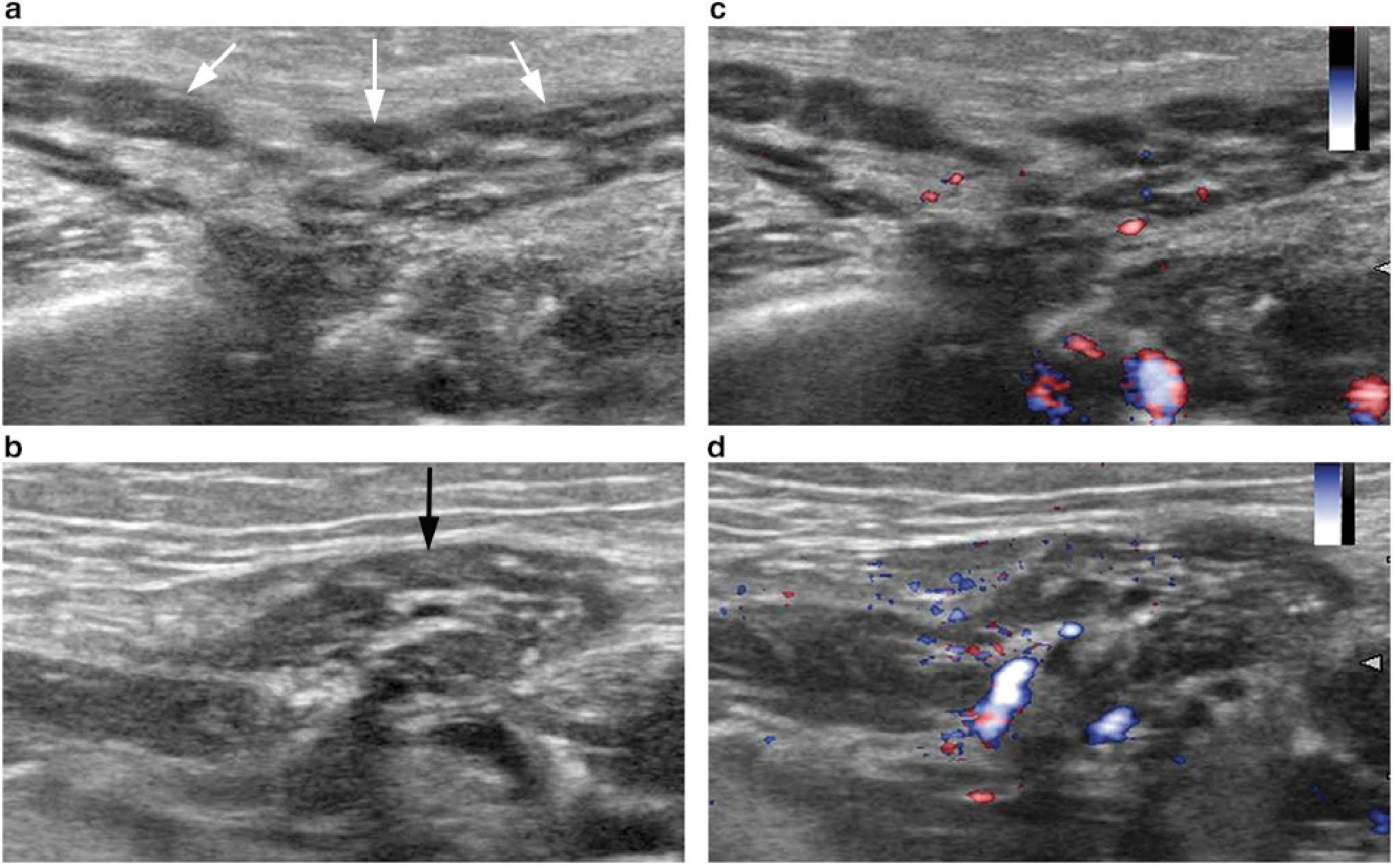
**a** Transverse B-mode ultrasound (US) images of the left parotid gland showing multiple ovoid hypoechoic spots with hyperechoic bands (white arrows). **b** Transverse B-mode US image of the left submandibular gland showing atrophy and diffusely distributed hyperechoic areas including small hypoechoic spots (black arrow). **c** Transverse fine-blood flow color Doppler US image of the left parotid gland showing no significantly increased vascularity. **d** Transverse fine-blood flow color Doppler US images of the left submandibular gland showing no significant increased vascularity

US was conducted using a HI VISION Preirus (Hitachi Medical Corp., Tokyo, Japan) with a 5–18 MHz linear array transducer (EUP-L75). B-mode images showed atrophic and heterogeneous changes in both the parotid and submandibular glands (Fig. 3a, b). Fine-blood flow color Doppler images did not show increased vascularity suggesting acute sialadenitis (Fig. 3c, d).

**Fig. 4:**
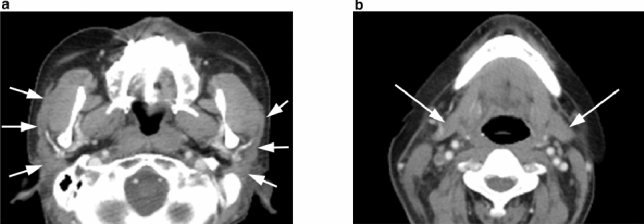
**a** Axial contrast-enhanced computed tomography (CT) image showing the atrophic changes with slightly higher than normal density of **a** parotid (white arrows) and **b** submandibular glands (white arrows). The CT images revealed no obvious heterogeneity in each salivary gland

Contrast-enhanced CT was conducted using an Ingenuity Elite 128-slice CT scanner (Philips Japan Ltd., Tokyo, Japan). CT images revealed atrophic changes and a density that was slightly higher than normal in both the parotid and submandibular glands; as shown on US, marked heterogeneity was not observed in each salivary gland (Fig. 4).

**Fig. 5:**
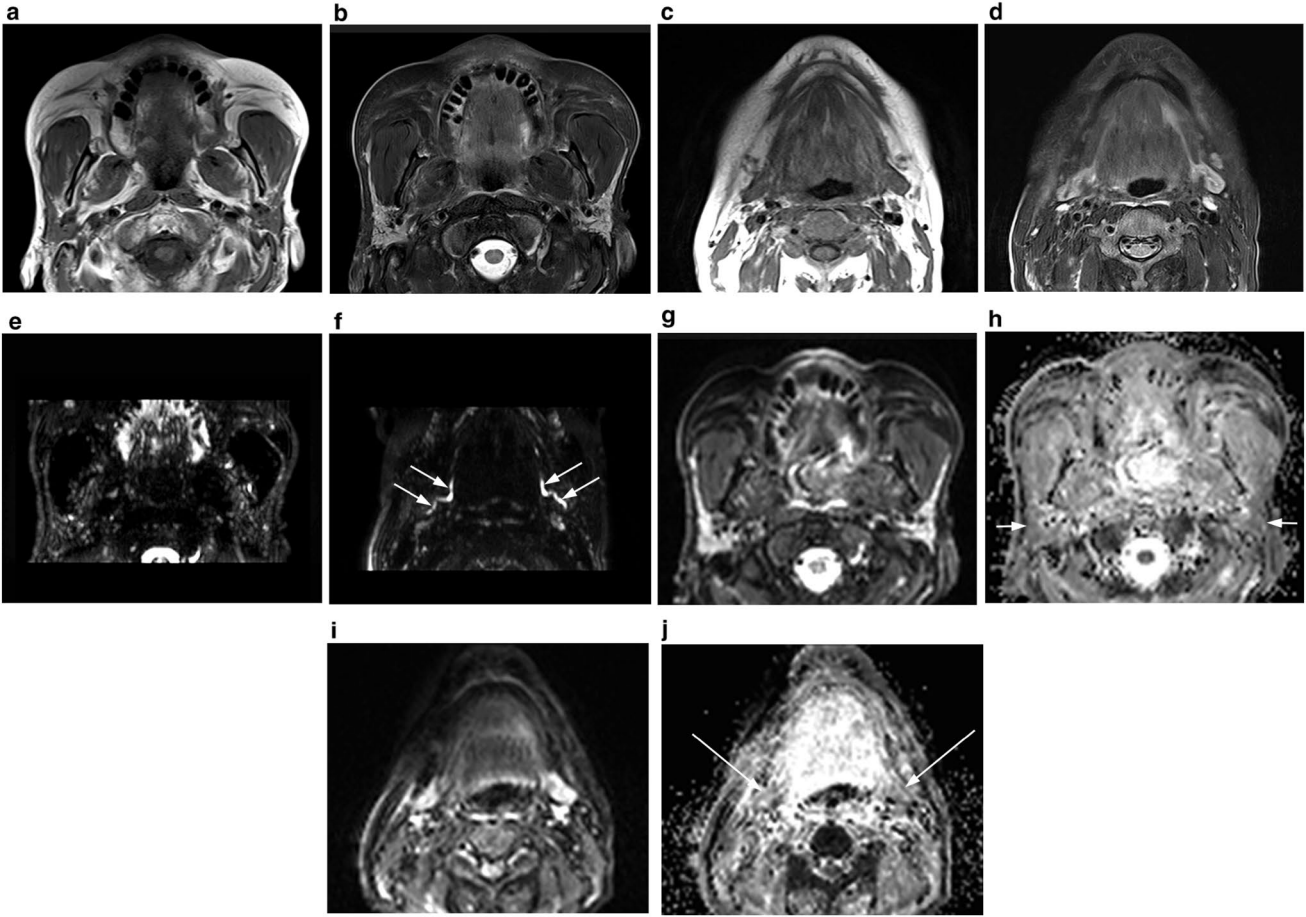
**a** Axial *T*_*1*_-weighted magnetic resonance imaging (MRI) showing the atrophic parotid glands with slightly lower signal intensity than normal. Most of the parenchyma in the glands were replaced by heterogeneous intermediate-intensity lesions with diffusely distributed small, low-intensity spots. **b** Axial *T*_*2*_-weighted fat saturation MRI showing the heterogeneous atrophic parotid glands with slightly lower signal intensity than normal. **c** Axial *T*_*1*_-weighted magnetic resonance imaging (MRI) of the submandibular glands and **d** *T*_*2*_-weighted fat saturation MRI of the submandibular glands showing the atrophic changes with slightly lower signal intensity than normal. **e** MR sialography of the parotid glands showing a few globular high-signal-intensity spots, although it does not show multiple globular high-signal-intensity spots in the glands and dilation with fluid retention of the Stensen’s duct. **f** MR sialography of the submandibular glands showing dilations with fluid retention of both Wharton’s ducts and intraglandular main ducts (white arrows). **g** Diffusion-weighted MRI of the parotid glands showing markedly high signal intensity as compared with that in the muscle and **h** apparent diffusion coefficient map of the parotid glands showing a markedly lower value than that in the muscle (white arrows). **i** Diffusion-weighted MRI of the submandibular glands showing marked high signal intensity as compared with muscle and **j** apparent diffusion coefficient map of the submandibular glands showing marked lower value than muscle (white arrows)

MRI was conducted using a MAGNETOM Prisma 3Tesla MRI scanner (Siemens K.K., Tokyo, Japan) with 64-channel head and neck coils. MRI demonstrated atrophic changes of both the parotid and submandibular glands, which had lower signal intensity than normal. The parotid gland was heterogeneous on *T*_*1*_-weighted images (*T*_*1*_WI) and *T*_*2*_-weighted images with fat saturation (*T*_*2*_WIfs), and it was replaced by multiple intermediate-signal lesions, including diffusely distributed small, low-signal spots. Moreover, the heterogeneous intermediate-intensity lesions and diffusely distributed small, low-intensity spots of *T*_*2*_WIfs corresponded to that of *T*_*1*_WI, respectively (Fig. 5a, b). By contrast, the submandibular glands did not reveal heterogeneity as much as the parotid glands on *T*_*1*_WI and *T*_*2*_WIfs (Fig. 5c, d). MR sialography showed a few globular high-signal spots of the parotid glands (Fig. 5e) and dilations of both Wharton’s duct and intraglandular main duct of the submandibular gland (Fig. 5f). Diffusion-weighted image (DWI) and apparent diffusion coefficient (ADC) map showed salivary glands with a heterogeneous marked high signal intensity and a lower value than normal, respectively (Fig. 5g–j).

## Discussion

The clinical characteristics of patients with xerostomia induced by ICI, such as nivolumab, are similar to those of SS patients, but the prevalence of SS-associated immunological markers such as antinuclear antibodies or anti-SSA in patients with ICI-induced xerostomia is low compared with patients with SS-induced xerostomia [6–8]. Furthermore, it has been reported that the immunohistochemistry lymphocyte profile is different between these conditions, and the B-cell ratio of ICI-induced xerostomia is obviously lower than that of SS-induced xerostomia [7, 8]. Our patient also demonstrated clinical and immunological characteristics similar to ICI-induced xerostomia. By contrast, several radiologic imaging findings of our patient differed from those of SS patients. Therefore, the examination of radiologic imaging features in this condition would further reveal the mechanism or cause of salivary gland hypofunction by ICI.

SS-induced xerostomia is characterized by a heterogeneous distribution of high signal intensity and low signal intensity (referred to as “salt and pepper appearance” on *T*_*1*_WI and *T*_*2*_WIfs [11]) and multiple high-signal-intensity spots with enlarged ducts called “apple tree appearance” on MR sialograph [12]. By contrast, in our patient, *T*_*1*_WI and *T*_*2*_WIfs of the salivary glands did not reveal findings such as SS, and the parotid glands of our patient showed multiple intermediate-signal lesions suggesting lymphocyte infiltration and small low-signal spots suggesting fibrosis, respectively. The histopathological glandular feature of ICI-induced xerostomia was lymphocytic sialadenitis [7, 8], and as the sialadenitis progresses, the more significant the degree of lymphocyte infiltration becomes [8]. As a result, these MRI findings might reflect the histopathological feature of severe ICI-induced xerostomia. Therefore, we believe that these MRI findings are important key points that can be used to distinguish between ICI-induced xerostomia and SS-induced xerostomia. In our patient, DWI and the ADC map showed high signal intensity and lower value compared with normal salivary glands, respectively. Similar findings were also indicated by the advanced stage of SS-induced xerostomia [13, 14], and these MRI findings have been suggested to reflect the decreased mobility of water from the reduction in salivary production caused by damage to the acinar tissues or narrowing of the intercellular space due to advanced lymphocytic infiltration [14]. Therefore, DWI and the ADC map of our patient were also consistent with the imaging findings of ICI-induced xerostomia.

The US findings of our patient showed multiple ovoid hypoechoic spots with hyperechoic bands in the parotid gland and diffusely distributed hyperechoic areas, including small hypoechoic spots in the submandibular gland. Cappelli et al. [6] and Warner et al. [8] reported similar US features of the parotid and submandibular glands in ICI-induced xerostomia. However, these US findings are also commonly seen in SS-induced xerostomia [15, 16]. In their study, Warner et al. [8] speculated a higher focus score on the labial salivary gland biopsy specimen indicating an increased number of and larger hypoechoic spots on US in ICI-induced xerostomia. Therefore, the hypoechoic spots on US in ICI-induced xerostomia might indicate a mass of lymphocyte infiltrations rather than retention of saliva, as with MRI.

In our patient, the findings of contrast-enhanced CT revealed atrophic changes and a homogeneous, slightly higher density lymph node in the parotid and submandibular glands. Typical CT findings of SS are heterogeneity, abnormal diffuse fat tissue deposition, and diffuse punctate calcification [17]. The CT findings of our patient suggest that typical CT findings of ICI-induced xerostomia may be different from those of SS-induced xerostomia. Moreover, the change in density of the parotid and submandibular glands in our patient might reflect the histopathological features of ICI-induced xerostomia, because these altered densities were similar to that of the lymph node.

On the basis of the radiologic imaging features of our patient, we found that MRI and US may be suitable for detecting glandular changes induced by ICI. Moreover, MRI may be the best imaging examination to distinguish ICI-induced xerostomia from SS-induced xerostomia. Furthermore, we hypothesize that the hypofunction of salivary gland in ICI-induced xerostomia is not caused by the destruction of the salivary glands with leaked saliva or enlarged ducts, which is commonly seen in SS-induced xerostomia, but rather by acinar cell damage due to diffusely spread lymphocyte infiltration throughout the salivary gland. Labial salivary gland biopsy specimens of severe ICI-induced xerostomia demonstrated by Warner et al. [8] also presented diffusely spread T-cell lymphocyte infiltration throughout the salivary gland. Therefore, there seems to be no major contradiction to our hypothesis about salivary gland hypofunction in ICI-induced xerostomia. However, to confirm our hypothesis, the radiologic imaging features of ICI-induced xerostomia in a larger number of cases need to be investigated.
